# An Orthotopic Patient-Derived Xenograft (PDX) Model Allows the Analysis of Metastasis-Associated Features in Colorectal Cancer

**DOI:** 10.3389/fonc.2022.869485

**Published:** 2022-06-28

**Authors:** Maria Laura De Angelis, Federica Francescangeli, Chiara Nicolazzo, Eljona Xhelili, Filippo La Torre, Lidia Colace, Alessandro Bruselles, Daniele Macchia, Sara Vitale, Paola Gazzaniga, Marta Baiocchi, Ann Zeuner

**Affiliations:** ^1^ Department of Oncology and Molecular Medicine, Istituto Superiore di Sanità, Rome, Italy; ^2^ Department of Molecular Medicine, Liquid Biopsy Unit, Sapienza University, Rome, Italy; ^3^ Surgical Sciences and Emergency Department, Policlinico Umberto I/Sapienza University of Rome, Rome, Italy; ^4^ Department of Surgical Sciences, Policlinico Umberto I/Sapienza University of Rome, Rome, Italy; ^5^ Center of Animal research and Welfare, Istituto Superiore di Sanità, Rome, Italy; ^6^ Department of Medicine and Traslational Surgery, Università Cattolica del Sacro Cuore, Rome, Italy

**Keywords:** patient-derived xenograft (PDX), colorectal cancer (CRC), metastasis, epithelial-to-mesenchymal transition (EMT), organoids

## Abstract

Metastasis is the primary cause of death in patients with colorectal cancer (CRC), urging the need for preclinical models that recapitulate the metastatic process at the individual patient level. We used an orthotopic patient-derived xenograft (PDX) obtained through the direct implantation of freshly dissociated CRC cells in the colon of immunocompromised mice to model the metastatic process. Ortho-PDX engraftment was associated to a specific set of molecular features of the parental tumor, such as epithelial-to-mesenchymal transition (EMT), TGF-β pathway activation, increased expression of stemness-associated factors and higher numbers of circulating tumor cells (CTCs) clusters expressing the metastatic marker CD44v6. A parallel analysis of orthotopic/metastatic xenografts and organoids showed that tumor cells underwent mesenchymal-to-epithelial transition at the metastatic site and that metastasis-derived organoids had increased chemotherapy resistance. These observations support the usefulness of ortho-PDX as a preclinical model to study metastasis-related features and provide preliminary evidence that EMT/stemness properties of primary colorectal tumors may be crucial for orthotopic tumor engraftment.

## Introduction

Colorectal cancer (CRC) is the second most frequent cancer worldwide (1). Despite the improvements in early diagnosis and therapy, its overall five-year lethality is 66% mostly due to synchronous or metachronous metastatic disease (1). For this reason, preclinical models for assessing the efficacy of antimetastatic agents are urgently needed. Unfortunately, murine CRC models display critical limitations in metastasis development. In fact, two frequently used murine models, i.e. chemically induced carcinogenesis and tumor-prone genetically engineered mouse models both poorly reproduce human cancer biology and pathogenesis. Regarding *in vivo* human CRC models, the approaches currently available are represented by xenografting human tumor fragments or cells into immunecompromised mice, either subcutaneously or orthotopically (2). Subcutaneous xenografts can be generated either from cell lines, patient tumors or primary CRC cells (cultivated either as spheroids or organoids). We and others have previously used spheroid-derived xenografts to investigate cancer-associated molecular pathways and mechanisms of therapy sensitivity (3–8). The subcutaneous implantation of freshly isolated tumor specimens (subcutaneous patient-derived xenografts, PDX) has an improved capability to preserve primary cancer genetic and cellular heterogeneity as compared to grafting of cultured cancer cells (9). Large panels of subcutaneous PDX have demonstrated a high prognostic and therapy predictive power in several studies (10–13). However, subcutaneous PDX do not generate metastases and therefore are unsuitable for anti-metastatic drug screening or efficacy prediction. In order to generate metastatic models, orthotopic grafting of CRC cells or tumor fragments is required (14). Orthotopic grafting has been executed mostly with poorly differentiated cell lines, thus limiting its clinical relevance (14). However, pioneer studies have performed orthotopic xenografts with mouse tumor organoids (15, 16), with human spheroids (17, 18) or organoids (19, 20) and with cells directly derived from human colorectal tumors (21–26). In this study, we generated an orthotopic PDX with CRC cells isolated from a surgical CRC specimen and directly transplanted into the colon of immunocompromised mice. Prompt grafting at primary site followed by metastatization was observed for cells from one out of three patients. Ortho-PDX gave rise to spontaneous lung and liver metastases and were employed to generate orthotopic-derived and metastasis-derived organoids, which were analyzed for EMT markers and chemoresistance. Interestingly, tumor cells that produced orthotopic/metastatic PDX were characterized by enhanced expression of stemness-related genes and of proteins involved in epithelial-to-mesenchymal transition (EMT), indicating an increased tumor aggressiveness. Finally, a characterization of patients’ circulating tumor cells (CTCs) showed increased numbers of CTCs and CTC clusters associated to ortho-PDX engraftment. Altogether, these results support ortho-PDX as a faithful model of metastatic CRC and provide preliminary evidence that the combination of stemness- and EMT-related features may promote orthotopic engraftment.

## Materials and Methods

### Patient-Derived Xenograft Generation 

Surgical specimens of colorectal cancer (CRC) were obtained upon informed consent from CRC patients undergoing surgery for primary tumor resection. Sample collection was performed under the approval of the Sapienza-Policlinico Umberto I Ethical Committee (RIF.CE: 4107 17/10/2016). Samples were washed 3 times in cold phosphate buffered saline (PBS) and transferred to Dulbecco’s modified Eagle’s medium (DMEM; Thermo Fisher, Foster City, CA, USA) containing 3% penicillin-streptomycin-amphotericin B solution (Thermo Fisher) until processing. Then, CRC samples were washed in PBS and manually cut in fragments > 0.5 mm that were subsequently incubated in Tryple Express (Thermo Fisher) for 30 min at 37 °C with shaking. The resulting suspension was filtered with a 100 μm nylon mesh, washed twice with DMEM and resuspended in Matrigel^®^ (Growth Factor Reduced Basement Membrane Matrix Corning, New York, USA) for orthotopic injection. Animal procedures were executed in accordance to the National animal experimentation guidelines (D.L.116/92) upon approval by the Animal Experimentation Committee of the Italian Ministry of Health (DM n. 292/2015 PR 23/4/2015). NOD.Cg-Prkdcscid Il2rgtm1Wjl/SzJ (NSG) mice (The Jackson Laboratory) (6-week-old females) were used for all *in vivo* experiments. Before injection, mice were anesthetized with ketamine (100 mg/kg) and xylazine (10 mg/kg), then 10^5^ cells resuspended in 40 μl 1:1 PBS/Matrigel were injected in the colon wall during open laparotomy. Animals were euthanized according to the national Animal Welfare Guidelines when they lost more than 20% of their body weight or alternatively (in case they did not display any sign of suffering) after 120 days from xenografting. Histological evaluations were performed by an expert pathologist.

### Generation and Validation of Xenograft-Derived Organoids

Organoid cultures were generated from orthotopic xenografts (OXDOs) or from metastatic xenografts (MXDOs) with the method described in (27). Shortly, cells were resuspended in Matrigel^®^ and seeded in 24 well plates. Cancer cells were overlaid with 500 µL of colon cancer organoids culture medium (27) supplemented with 20 ng/mL recombinant human EGF, 10 ng/ml human basic fibroblast growth factor (both from Peprotech, Rochy Hill, NJ, USA), 10 nM Gastrin, 10 µM Y-27632, 10 µM SB202190 (Sigma-Aldrich, St. Louis, MO) and 500 nM A83-01 (Tocris Bioscience, Bristol, UK).

### CTCs Isolation From the Peripheral Blood of CRC Patients

Peripheral blood samples were obtained from three patients with occlusive CRC according to the protocol approved by Ethical Committee of Policlinico Umberto I of Rome (protocol n. 668/09, July 09, 2009; amended protocol 179/16, March 01, 2016). Each sample was collected into K2EDTA tube, stored at +4°C and processed within 3hrs. In order to isolate CTCs for cytological studies, the ScreenCell^®^ Cyto kit (ScreenCell, Sarcelles, France) was employed following the manufacturer’s instructions.

### Mutational Profiling

Genomic DNA was extracted from tumor tissues with the DNeasy Mini Kit (Qiagen, Limburg, The Netherlands) and used for mutation analysis. Data analysis on tumor samples was carried out using the Ion Reporter Software v5.12 (Thermo Fisher Scientific) following AmpliSeq CHPv2 single sample workflow which detects and annotates low frequency variants (SNPs, InDels). All the selected variants were visually inspected using the Integrative Genomics Viewer (IGV; www.broadinstitute.org/igv).

### Immunohistochemistry

Tissues were fixed in paraformaldehyde 4%. Fixation was followed by dehydration, embedding in paraffin, section cutting, and standard H&E staining. For immunohistochemistry staining, samples were incubated with primary antibodies anti-CK20, anti-ZEB2 (#M7019, Dako, Agilent Technologies; #NBP1-82991, Novus), anti-alphaSMA (Cell Signaling mAb #19245), anti pSMAD 2/3 (Invitrogen #PA5-110155) and anti-FAP (Cell Signaling mAb #66562). The sections were subsequently incubated with secondary antibodies and visualised using the UltraTek HRP Anti-Polyvalent DAB (Scytek). Nuclei were counterstained with Mayer’s haematoxylin. IHC on liver sections was performed with previous block of endogenous biotins. Images were acquired on a Zeiss Axio Scope.A1 Microscope equipped with 20X objectives and quantified with the software ZEN 2.6 (blue edition).

### Real-Time PCR

Total RNA was extracted with TRIzol (Thermo Fisher) and reverse-transcribed with M-MLV reverse transcriptase (Thermo Fisher). The resulting cDNA was used as template in PCR reactions with the following probes: Vimentin (Hs.PT.58.38906895), E-Cadherin (Hs.PT.58.3324071), ZEB1 (Hs.PT.58.39178574), ZEB2 (Hs.PT.58.1089006), SNAIL (Hs.PT.58.2984401), SLUG (Hs.PT.58.1772559), TWIST (Hs.PT.58.18940950), Bmi-1 (Hs00180411_m1), NANOG (Hs.PT.58.21480849), Lgr5 (Hs00969422_m1), all from Integrated DNA Technologies, Coralville, USA). Normalization was performed using β-ACTIN (Hs.PT.39a.22214847) as reference. Values were expressed as 2-ΔΔCt, where ΔΔCT = ΔCTsample−ΔCTcalibrator or ΔCt. ΔCt represents the difference in threshold cycles between specific RNA and reference amplicons provided by StepOne Plus Real-Time PCR software upon negative correlation with the internal reference dye (ROX).

### Enzyme-Linked Immunosorbent Assay

TGF-β protein levels were evaluated in lysates of tumors and normal mucosae of CRC patients by using DuoSet^®^ ELISA kit and DuoSet^®^ ELISA Ancillary Reagent Kit 1 (both from R&D Systems, Minneapolis, MN) according to the manufacturer’s recommendations. To activate latent TGF-β1 to its immunoreactive form, samples were first incubated with Sample Activation Kit 1 (R&D Systems, Catalog # DY010). The relative absorbance was read at 490 nm on a Benchmark microplate reader (Bio-Rad Laboratories).

### Western Blotting

Pieces of frozen tissues (~ 50 μg) were lysed in lysis buffer (10 mM Tris pH8, 150 mM NaCl, 60 mM Octyl-β-Glucoside, supplemented with protease inhibitor cocktail/phosphatase inhibitor cocktails I and II from Sigma-Aldrich). Tissue homogenization was performed with Pro 200 Kema Keur (Pro Scientific Inc. Oxford) at max speed at 4°C for 30”. Equivalent amounts of proteins were loaded on 4–12% precast gels (Thermo Fisher) and transferred onto nitrocellulose membranes (GE Healthcare Life sciences). Blots were incubated first with TBST 5% nonfat dry milk and then overnight at 4°C with primary antibodies. Vimentin (#5741), N-Cadherin (#13116), SLUG (#9585), ZEB1 (#3396), pSMAD 2/3 (#8828), TGFβ (#3711) were from Cell Signaling Technology, E-Cadherin (#610181) from Becton Dickinson, LTBP1 from Santa Cruz (#sc-271140). Blots were then washed 4 times in TBST and incubated for 45 minutes with secondary HRP-conjugated antibodies diluted in TBST 5% nonfat dry milk. Immunoblotting images were recorded and analyzed with Bio-Rad ChemiDoc Imagers (Bio-Rad Laboratories). Immunoblot densitometry quantification was performed with ChemiDocMP (BioRad) and signal intensity was quantified with the Image Lab software. Normalization was performed using antibodies against β-ACTIN or GAPDH as reference standards (#A5316, #SAB1405848 respectively, from Sigma-Aldrich).

### Immunofluorescence Staining of CTCs

For immunofluorescence analyses, CTC isolation filters were hydrated with Tris-Buffered Saline (TBS) and first stained with mouse monoclonal anti-human CD45 (#130–098-551, Miltenyi Biotec) in order to detect hematopoietic cells. Then, filters were washed twice in TBS 0.002% Tween20 and endogenous peroxidase activity was neutralized by incubating with 0.03% hydrogen peroxide for 15 min in the dark, followed by incubation for 90 minutes with CD45 biotinylated antibody. Filters were then processed using streptavidin conjugated to horseradish peroxidase and substrate-chromogen solution contained in UltraTek HRP Anti-Polyvalent DAB kit (#AMF080, Scytek). Samples were then incubated in a humid chamber overnight at 4°C with the following primary antibodies: anti-Vimentin (#5741, Cell Signaling), anti-CK20 (#SC-17113, Santa Cruz Bio-technology) and anti-CD44v6 (#BBA13, R&D Systems). Filters were washed and incubated with appropriate secondary antibodies (Donkey anti-rabbit IgG Alexa Flu-or^®^488-conjugated #A21206, donkey anti-goat IgG Alexa Fluor^®^647-conjugated #A21447, donkey anti-mouse IgG Alexa Fluor^®^555-conjugated #A-31570) for 45 minutes at RT in the dark. Nuclei were stained with 4′, 6-Diamidino-2-Phenylindole (DAPI #D1306, Thermo Fisher) for 15 minutes at RT. All antibodies were dissolved in PBS 3% bovine serum albumin (BSA), 3% fetal bovine serum (FBS), 0.001% NaN_3_ and 0.1% Triton X-100. Finally, filters were mounted with Prolong-Gold Antifade (#P7481, Thermo Fisher) on glass slides and analyzed using an Olympus FV1000 confocal microscope equipped with 60x oil immersion objectives.

### Viability Assay

Organoids were dissociated into single cells and plated in 30μl 1:1 Medium/Matrigel in 96 well plates (3,500 cells/well) in triplicate for 72 hours prior to drug treatment. Organoids were treated for 6 days with 5-Fluorouracil (Selleck Chemicals) in a humidified atmosphere at 37°C, 5% CO2 and drug-containing medium was replaced every 72 hours. Cell viability was determined by CellTiter Glo 3D viability assay (Promega) with a DTX880 multimode microplate reader (Beckman Coulter).

### Migration/Invasion Assay

4 x 10^3^ cells obtained from dissociated OXDOs or MXDOs were allowed to re-aggregate into organoids for 4 days and then plated in Matrigel^®^ into the upper wells of Boyden Chambers containing porous 8 mm diameter polycarbonate membranes (Costar Scientific Corporation) suspended in 200 μl of non-supplemented organoid medium. Lower wells contained 500 μl of organoid medium supplemented with 20 ng/ml EGF and 10 ng/ml basic FGF. After 72 hours, cells in the upper wells were removed, whereas cells that migrated to the lower wells were fixed in 4% PFA, stained with DAPI in PBS 1% NP40 for 5 min and counted under a fluorescence Zeiss Axio Scope.A1 Microscope equipped with a 10x objective. The number of migrated cells was quantified with the ZEN 2.6 software (blue edition).

### Statistical Analysis

Statistical analyses were performed using GraphPad Prism version 4.0 for Windows (GraphPad Software) with unpaired Student’s t test. Results are presented as the mean ± SD or mean ± SEM where appropriate. Statistical significance is expressed as *, P < 0.05, **, P < 0.01 and ***, P < 0.001.

## Results

### Establishment of Orthotopic/Metastatic Patient-Derived Xenografts: Workflow of the Study

**Figure 1:**
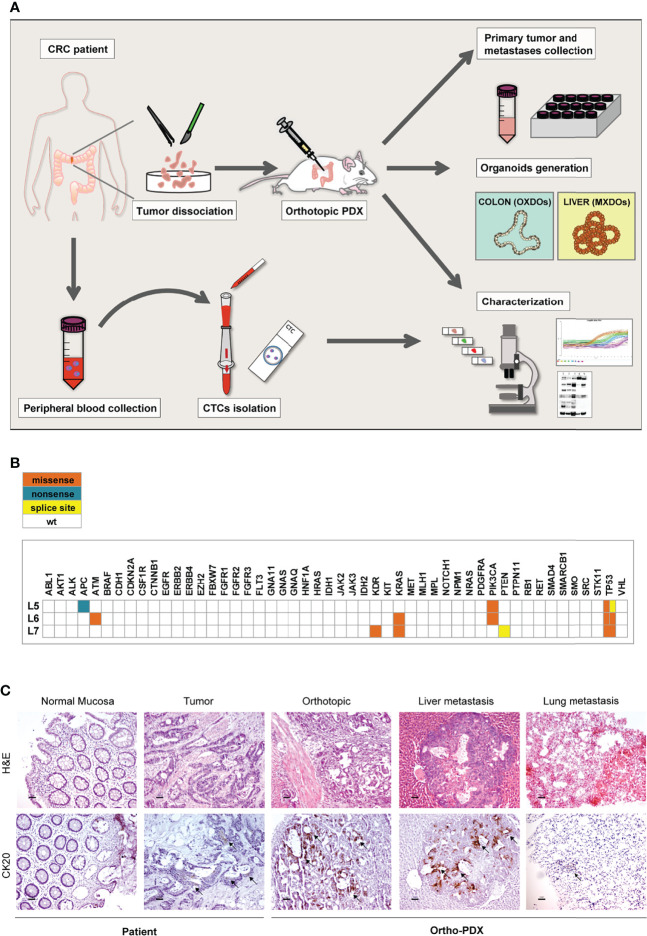
Workflow of the orthotopic PDX model. **(A)** Orthotopic patient-derived xenografts (Ortho-PDX) were directly generated from a colon adenocarcinoma surgically removed from a CRC patient. Dissociated tumor cells were injected in the colon wall of immunocompromised (NSG) mice. Upon tumor formation, intestinal and metastatic tumor tissues were collected and characterized. Organoid were generated from orthotopic xenografts (orthotopic xenograft-derived organoids, OXDOs) and from liver metastases (metastatic xenograft-derived organoids, MXDOs). Circulating tumor cells (CTCs) were collected from patients’ peripheral blood, counted and analyzed for marker expression. **(B)** Distribution of functionally relevant variants found among the 50 genes included in the Ion AmpliSeq Cancer Hotspot Panel v2 panel, in the 3 samples under study. Missense, nonsense and splice site variants are depicted in orange, blue and yellow, respectively. **(C)** Paraffin-embedded sections of normal mucosa, patient tumor, orthotopic tumor, liver and lung metastases were stained with Hematoxylin/Eosin (H&E, upper panels) and cytokeratin-20 (CK20, lower panels). Magnification 20x. Bar 50 μm.

We have previously described a metastatic CRC model based on the orthotopic injection of stem cell-enriched multicellular spheroid cultures into the colon wall of immunocompromised mice, giving rise to liver and lung metastasis. In our hands, this method demonstrated an efficacy of about 70-80% of colon grafting/liver metastasis (17, 18). In order to extend the orthotopic/metastatic CRC model to patient-derived xenografting, we inoculated freshly dissociated cells obtained from tumor tissues of three patients, (referred to as L5, L6, L7) into the colon of NSG mice (10^5^ cells/graft, 5 replicate mice/patient) according to the workflow shown in **Figure 1A**. Inoculation of dissociated tumor cells was preferred over the implantation of tumor fragments for quantitative reasons, as it allowed to inoculate the same number of cells for each patient. Furthermore, as orthotopic transplantation requires engraftment of tumor cells into in the mouse colon, this technique is more practicable (and less distressful for animals) by performing cell injection in the cecum wall rather than through sawing a tumor fragment in the colon lumen. Furthermore, this technique was previously validated in CRC orthotopic xenografting (24). Clinical data and microsatellite stability data are provided in **Supplementary Figure 1A**, patients’ mutational profile is reported in **Figure 1B**. Tumor histology is shown in **Figure 1C** and **Supplementary Figure 1B**. Mice were sacrificed when presenting ~20% loss of body weight or alternatively (in case they did not display any sign of suffering) after 120 days from xenografting, and examined for the presence of tumors into the colon and at distant organs. Xenografts performed with CRC patients L5 and L7 did not give rise to orthotopic or metastatic tumors. By contrast, xenografts performed with CRC patient L6 gave rise to orthotopic tumors and metastases in both lungs and liver in 4/5 mice. Ortho-PDX and metastases were harvested and stained with Hematoxylin/Eosin and cytokeratin 20 (CK20). Histological evaluation and CK20 staining of orthotopic tumors and of the deriving hepatic and pulmonary metastases confirmed their CRC origin (**Figure 1C**). One orthotopic tumor xenograft derived from patient L6 was dissociated into single cells and cultured to generate organoids (orthotopic xenograft-derived organoids, OXDOs). The same procedure was applied to liver metastases in order to generate metastatic xenograft-derived organoids (MXDOs). Lung metastases were not sufficiently large to allow generation of organoid cultures.

### Ortho-PDX Generation Is Associated With a Hybrid Epithelial-Mesenchymal Phenotype of the Parental Tumor

**Figure 2:**
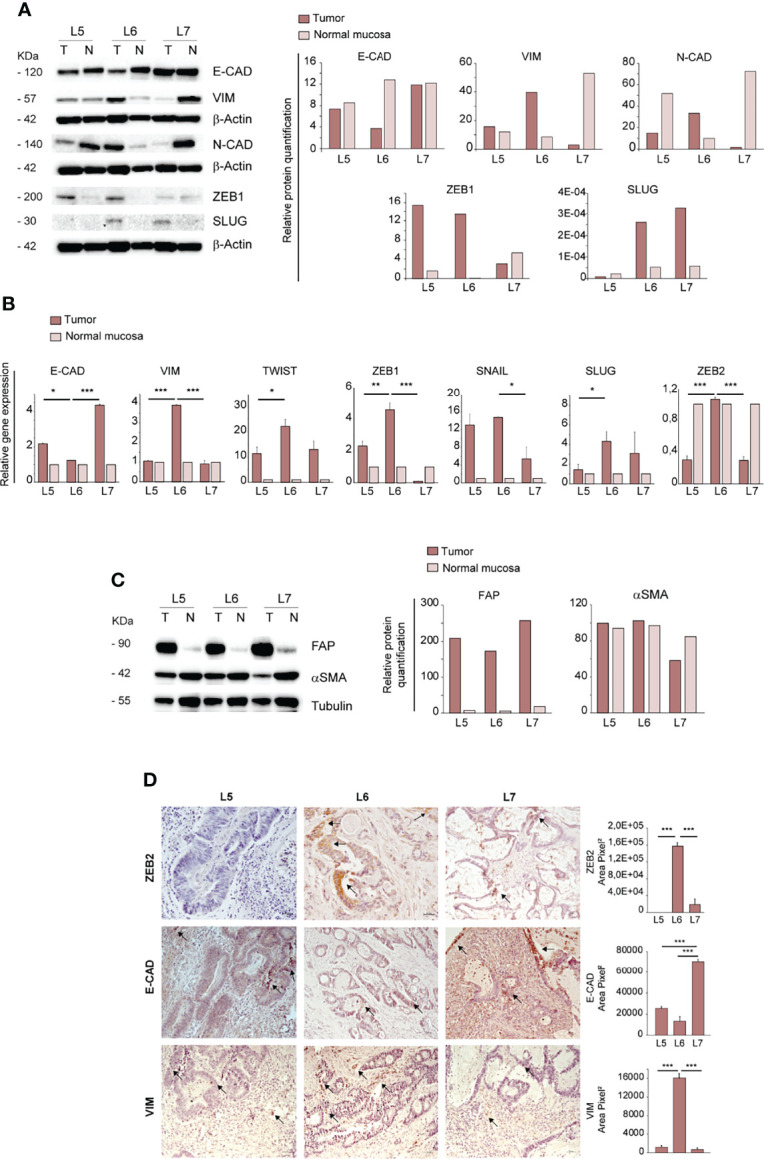
Ortho-PDX generation is associated with a mesenchymal phenotype of the parental tumor. **(A)** Left: immunoblot analysis of E-Cadherin, Vimentin, N-Cadherin, ZEB1 and SLUG on whole lysates of normal mucosa (N) and patient tumors (T). β-Actin was used as a loading control. Right: quantification of the immunoblot experiment shown on the left. **(B)** qRT-PCR analysis of Vimentin, Twist, E-Cadherin, ZEB1, SNAIL, SLUG and ZEB2 expression in normal mucosa and tumor tissue of patient tumors L5, L6, L7. Mean ± SD of 3 experiments. *P < 0.05, **P < 0.01 and ***P < 0.001 from two-tailed t test. **(C)** Left: Immunoblot analysis of Fibroblast Activation Protein (FAP) and alpha Smooth Muscle Actin (αSMA) on normal mucosa (N) and patient tumors (T), with tubulin as loading control. Right: quantification of the immunoblot experiment shown on the left. **(D)** Left: Paraffin-embedded sections of patient tumors L5, L6, L7 were stained with anti-ZEB2 (upper panels), anti-E-Cadherin (E-CAD, central panels) and anti-Vimentin (VIM, lower panels). Arrows show areas positive for protein expression. Right: quantification of ZEB2, E-CAD and VIM performed on patients L5, L6, L7, 5 fields/section. Magnification 20x. Bar 50 μm.

Having established that only the CRC derived from the L6 patient was able to generate ortho-PDX and metastases, we investigated whether orthotopic engraftment was associated to specific cellular and molecular features of the parental tumor. In particular, we analyzed a panel of factors associated to an epithelial or mesenchymal state, as EMT is considered a paradigm of tumor aggressiveness and stemness (28). To this end, we compared protein levels of E-Cadherin, Vimentin, N-Cadherin, ZEB1 and SLUG in paired normal mucosa/tumor samples derived from L5, L6 and L7 patients. E-Cadherin levels were lower in L6 tumor as compared to both normal tissues and tumor tissues from L5 and L7 patients, indicating weaker epithelial features in L6. By contrast, L6 showed a higher expression of EMT-associated markers Vimentin and N-Cadherin as compared to other tumors and elevated (although not highest) expression of ZEB1 and SLUG (**Figure 2A**). Then, we analyzed RNA expression of epithelial and mesenchymal-associated factors in normal and neoplastic tissues of L5, L6 and L7 patients. Also at the RNA level, we detected a lower expression of E-Cadherin and a higher expression of EMT-associated factors Vimentin, TWIST, ZEB1, SNAIL, SLUG and ZEB2 in L6 as compared to the other patients’ tumors (**Figure 2B**). Notably, the difference in ZEB2 RNA expression between L6 and the other tumors was highly significant, according to our recent finding that ZEB2 is associated with tumor stemness and EMT in CRC (7). Then, we asked whether a difference in the amount of tumor stroma was present in parental L5, L6 and L7 tumors, which could influence the expression levels of mesenchymal markers and possibly the success of orthotopic engraftment. To this end, we compared the expression of stromal markers Fibroblast Activation Protein (FAP) and alpha-Smooth Muscle Actin (αSMA) in the three patients. Immunoblot analysis and quantification of the results however showed that the three patients had comparable levels of stromal markers (**Figure 2C**), indicating that the differences in EMT factors expression can be attributed to tumor cells. Finally, we performed immunohistochemistry on L5, L6 and L7 tumor sections to analyze the expression of ZEB2 (for which we could not find an efficient antibody batch for immunoblotting), E-Cadherin and Vimentin respectively in tumor and stromal cells. In line with RT-PCR results, we found that ZEB2 was highly expressed in L6 tumor cells, while L5 was completely negative and L7 showed only few positive tumor cells (**Figure 2D**, upper panels). L6 had the lowest E-Cadherin expression and the highest Vimentin expression among the three tumors (**Figure 2D**, central and lower panels). ZEB2 and Vimentin staining in L6 were clearly located in tumor pseudocrypts, further supporting a more pronounced EMT state of tumor cells. Although ortho-PDX generation was performed with a small number of patients, these results suggest that a hybrid epithelial/mesenchymal state of patient’s CRC may be associated to a successful engraftment of orthotopic tumors. Finally, as EMT weakens the adhesion forces between tumor cells and promote independent or collective migration, we analyzed the numbers and features of circulating tumor cells (CTCs) in L5, L6 and L7 patients. L6 had an increased presence of CTCs and particularly of CTC clusters, which have been shown to be associated with increased metastatic capacity (**Supplementary Figure 2A**) (29, 30). Representative images of CTCs isolated from the peripheral blood of L5, L6 and L7 patients with the ScreenCell^®^ method show the presence in L6 of small CTCs clusters expressing high levels of Vimentin and CD44v6 (**Supplementary Figure 2B**). The latter was previously reported to characterize metastatic CSCs in CRC (31) and its expression on CTCs has been recently associated with treatment failure in metastatic CRC (32).

### TGF-β Pathway Activation and Increased Expression of Stemness-Associated Factors May Be Related to Orthotopic Engraftment

**Figure 3:**
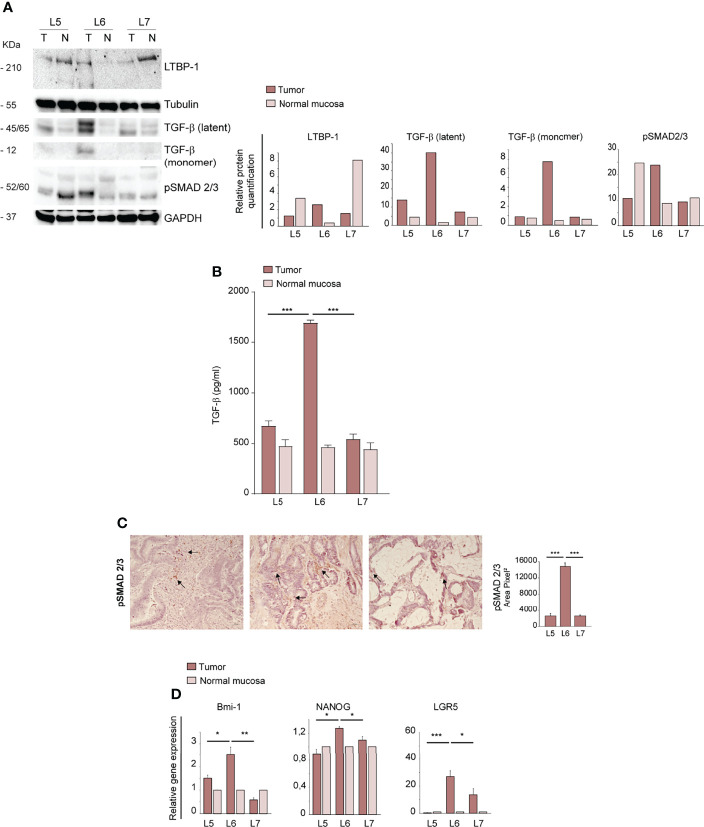
Ortho-PDX generation is associated with TGF-β pathway activation and increased expression of stemness-associated factors in the parental tumor. **(A)** Left: immunoblot analysis of TGF-β, LTBP-1 (Latent-transforming growth factor beta-binding protein 1), Latent TGF-β, active TGF-β and phosphorylated SMAD 2/3 (pSMAD 2/3) on whole lysates of normal mucosae (N) and patient tumors (T). Tubulin and Glyceraldehyde-3-phosphate dehydrogenase (GAPDH) were used as loading controls. Right: quantification of the immunoblot experiment shown on the left. **(B)** ELISA assay performed on lysates of normal mucosae (N) and patient tumors (T) showing active TGF-β concentration. ***P < 0.001 from two-tailed t test. **(C)** pSMAD 2/3 staining of L5, L6 and L7 tumor sections and quantification of 5 fields/section. Magnification 20x. Bar 50 μm. **(D)** qRT-PCR analysis of Bmi-1, Nanog and LGR5 expression in normal mucosae and tumor tissues of CRC patients. Mean ± SD of 3 experiments. *P < 0.05, **P < 0.01 and ***P < 0.001 from two-tailed t test.

As TGF-β is a major inducer of EMT (33, 34), we analyzed the expression of mature TGF-β and its precursor proteins and the levels of phosphorylated SMAD 2/3 (pSMAD 2/3) in normal intestinal mucosae and tumors derived from L5, L6 and L7 patients. The large latent TGF-β complex protein LTBP1 (Latent Transforming Growth Factor beta 1 binding protein), which participates in the local regulation of TGF-β signaling and in TGF-β tissue storage, had a slightly increased expression in L6 as compared to L5 and L7 tumors. The 45/65 kDa latent TGF-β and the 12,5 kDa mature monomer were highly expressed in L6, suggesting an activated state of this pathway (**Figure 3A**). Immunoblot analysis of pSMAD 2/3 showed a high expression both in L6 CRC and in L5 normal mucosa (**Figure 3A**). However, only L6 had both high active TGF-β and pSMAD 2/3 expression, indicating effective activation of TGF-β signaling. The increased levels of active TGF-β in L6 CRC as compared to other patients was confirmed by enzyme-linked immunosorbent assay (ELISA) performed on tumor lysates. TGF-β concentration in the L6 sample was significantly higher as compared to L5 and L7, in line with levels of TGF-β monomer detected by immunoblotting (**Figure 3B**). To gain further insight into the activation and location of TGF-β signaling, we performed IHC analysis of pSMAD 2/3 in L5, L6 and L7 tumor sections. The results showed that in L5 and L7 pSMAD 2/3 was localized respectively in stromal and tumor cells (**Figure 3C**). By contrast, L6 expressed significantly higher amounts of pSMAD 2/3 localized in tumor cells, further supporting the presence of active TGF-β signaling (**Figure 3C**). Since TGF-β signaling has been recently shown to induce de-differentiation and enhance stem cell properties in CRC (35), we analyzed transcript levels of factors implicated in stemness and self-renewal Bmi-1, Nanog and LGR5 in normal mucosae and tumors of L5, L6 and L7 patients. L6 CRC showed a significantly higher expression of the three factors, in line with its increased aggressiveness and mesenchymal features (**Figure 3D**). Altogether, these results suggest that an enhanced activation of EMT and stemness programs may promote tumors’ ability to generate orthotopic/metastatic PDX.

### Analysis of EMT- and Metastasis-Associated Features in Xenografts and Organoids Derived From Ortho-PDX and Liver Metastases

**Figure 4:**
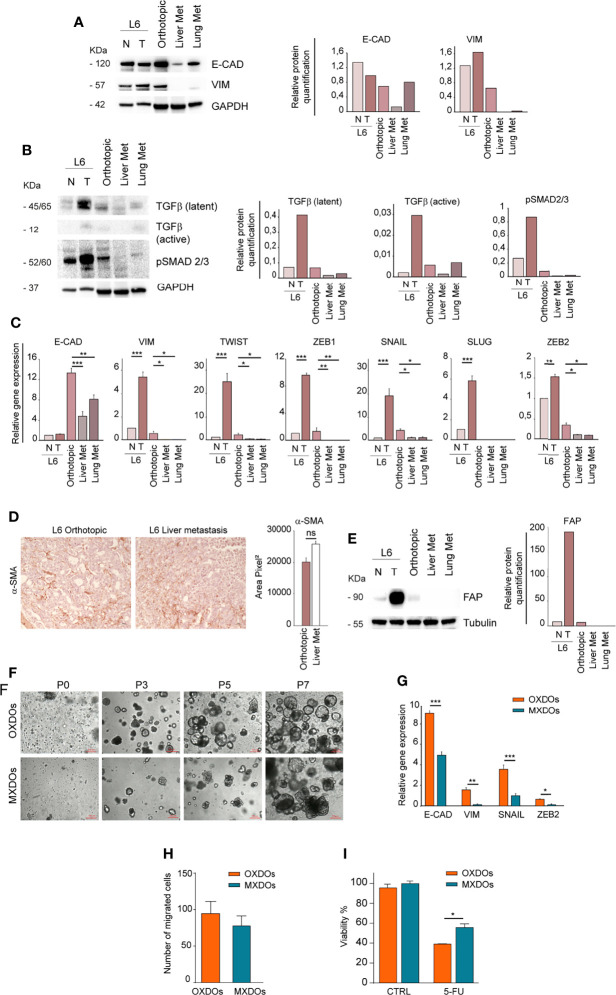
Metastatic tissues lose mesenchymal traits and acquire increased chemoresistance. **(A)** Left: comparative immunoblot analysis of E-Cadherin (E-CAD) and Vimentin (VIM) on whole lysates of normal/peritumoral mucosa (N) and tumor tissue (T) of patient L6, orthotopic xenograft (Orthotopic), liver and lung xenograft metastases (Liver Met and Lung Met). GAPDH was used as a loading control. Right: quantification of the immunoblot experiment shown on the left. **(B)** Left: comparative immunoblot analysis of phosphorylated SMAD 2/3 (pSMAD 2/3) and TGF-β on whole lysates as described above. Right: quantification of the immunoblot experiment shown on the left. **(C)** qRT-PCR analysis of E-Cadherin (E-CAD), Vimentin (VIM), Twist, ZEB1, SNAIL, SLUG, and ZEB2 in normal/peritumoral mucosa (N) and tumor tissue (T) of patient L6, orthotopic xenograft (Orthotopic), liver and lung xenograft metastases (Liver Met and Lung Met). Mean ± SD of 3 experiments. *P < 0.05, **P < 0.01 and ***P < 0.001 from two-tailed Student’s t test. **(D)** Staining of alpha Smooth Muscle Actin (αSMA) on sections of L6 orthotopic PDX and hepatic metastasis (left) and quantification of 5 sections (right). Magnification 20x, ns non-significant. **(E)** Left: Immunoblot analysis of human Fibroblast Activation Protein (FAP) in normal (N) and tumor (T) parental tissues of L6 patient, in orthotopic PDX (Orthotopic) and metastatic PDX (Liver Met and Lung Met). Tubulin was used as loading control. Right: quantification of the immunoblot experiment shown on the left. **(F)** Time course of organoid generation (orthotopic xenograft-derived organoids, OXDOs and metastatic xenograft-derived organoids, MXDOs), from the first day of culture (passage 0, P0) to subsequent passages (P3, P5 and P7, respectively after 3 weeks, 5 weeks and 7 weeks of culture). Magnification 10x. Bar 100 μM. **(G)** qRT-PCR analysis of E-Cadherin (E-CAD), Vimentin (VIM), SNAIL and ZEB2 on OXDOs (orange bars) and MXDOs (blue bars). *P < 0.05, **P < 0.01 and ***P < 0.001 from two-tailed Student’s t test. **(H)** Invasion/migration assay performed with OXDOs and MXDOs. **(I)** Cell viability of OXDOs (orange bars) and MXDOs (blue bars) treated with 5 μM 5-Fluorouracil (5-FU) for 6 days. Values represent mean ± SD of three independent experiments. *P < 0.05 by unpaired Student’s t test.

In order to investigate whether the different anatomic location of orthotopic and metastatic PDX affected the expression of EMT-associated factors, we first compared the expression of E-Cadherin and Vimentin in primary, orthotopic and metastatic tumor tissues. While E-Cadherin levels were variable with the highest expression in the normal mucosa, Vimentin was virtually undetectable in lung and liver metastatic tumors (**Figure 4A**). This result is in line with the mesenchymal-to-epithelial (MET) transition theory, indicating that tumor cells lose mesenchymal features once they have colonized metastatic sites. According to this hypothesis, orthotopic and metastatic tumor tissues expressed TGF-β but only the orthotopic tumor expressed a relevant amount of pSMAD 2/3, indicating activation of the TGF-β pathway (**Figure 4B**). RT-PCR analysis of EMT-associated factors showed that the orthotopic tumor had an increased expression of EMT-associated factors Vimentin, Twist, ZEB1, SNAIL and ZEB2 as compared to metastatic tissues (**Figure 4C**). E-cadherin levels were also highest in the orthotopic xenograft, possibly reflecting a hybrid epithelial/mesenchymal phenotype present also in the original patient tumor and peritumoral tissue (**Figure 4C**). An important issue is whether the parental tumor stroma is able to engraft and grow within mouse PDXs. Previous studies indicated that first generation PDXs contain both human and murine stroma, while after three PDXs generations the stroma is composed only by mouse cells (36). However, the large majority of PDXs studies were performed on subcutaneous xenografts, while information on orthotopic PDXs is limited. To characterize the relative amount of stroma in the orthotopic xenograft and in liver metastases we first performed an evaluation of αSMA (recognizing both human and mouse protein) on sections of orthotopic and metastatic PDXs (**Figure 4D**). IHC evaluation showed a non-significant difference between αSMA expression in the two tumor sites (**Figure 4D**). Then, we determined the presence of parental fibroblasts in the orthotopic PDX and in metastatic tumors by immunoblotting with anti-human FAP. The results shown in **Figure 4E** show that fibroblasts are abundant in the parental tumor but scarce in the ortho-PDX and completely absent from lung and liver metastases. Altogether, these observations suggest that parental tumor-associated stroma is not responsible for the EMT-associated features detected in L6. Patient-derived organoids (PDOs) obtained by subcutaneous transplantation of tumor fragments have been shown to reproduce the architectural and histological features of the original tumor tissue, and can be effectively used for the characterization/validation of molecular vulnerabilities for therapeutic purposes (37, 38). We generated organoids from orthotopic tumor xenografts (OXDOs) and hepatic metastases (MXDOs) from patient L6. OXDOs and MXDOs displayed similar growth rates (data not shown) and were regularly expanded until passage 7 (**Figure 4F**), then stored in liquid nitrogen and thawed as necessary. RT-PCR analysis of E-cadherin, Vimentin, SNAIL and ZEB2 expression in MXDOs reflected that of metastatic xenografts, with MXDOs showing a more epithelial state as compared to OXDOs (**Figure 4G**). Then, we performed a migration/invasion assay to compare this ability in OXDOs and MXDOs. OXDOs showed a slightly higher migratory capacity than MXDOs, but differences were not significant (**Figure 4H**, pictures in **Supplementary Figure 3**). Finally, we compared the chemosensitivity of OXDOs and MXDOs by treating organoids for 6 days with 5-fluorouracil (5-FU). MXDOs showed an increased resistance to 5-FU as compared to OXDOs, in line with the enhanced chemoresistance of metastatic tumors (**Figure 4I**).

## Discussion

Effective modelling of tumors through *in vitro* and *in vivo* methods is the cornerstone of preclinical cancer research, allowing dissection of cancer-associated molecular traits and testing of novel therapeutic strategies. In recent years, organoids and PDX have opened additional avenues towards personalized medicine, as they provide respectively an *in vitro* and *in vivo* reproduction of individual patient tumors. In this study, we provide an example of orthotopic-metastatic PDX directly generated from a CRC patient, which was used to study molecular features associated to primary, orthotopic and metastatic tumor tissues. Previous studies showed the feasibility of the ortho-PDX model in CRC. However, several studies did not examine in depth the molecular features of orthotopic and metastatic PDX (20–22, 24). In other cases, ortho-PDX transplantation was preceded by pre-conditioning through subcutaneous transplantation, thus selecting for cells more adaptable to a non-physiological microenvironment (23, 25, 26, 30, 39). In this manuscript, we provide further support to the feasibility of direct ortho-PDX generation and preliminary insights into mechanisms that may influence ortho-PDX engraftment. Interestingly, despite the small number of samples used in this study, we observed an association between ortho-PDX generation and EMT traits, increased activation of TGF-β signaling, expression of stemness-associated factors and presence of CTCs clusters in the peripheral blood. Our results are in line with recent studies showing that the EMT state may support metastatic seeding by CTCs clusters and that ZEB1 is required for liver metastatization in an orthotopic CRC model (30). Interestingly, our studies showed that ortho-PDX generation was associated to a particularly high expression of ZEB2 in primary tumor cells. ZEB2 is a transcriptional regulator linked to EMT and stem cell plasticity (40, 41). We have recently demonstrated that, in CRC, ZEB2 was associated to slow cycling, enhanced stemness, chemoresistance, mesenchymal features and worse relapse-free survival (7). Therefore, it can be hypothesized that tumors characterized by elevated ZEB2 expression, stem cell traits and EMT may be particularly suitable for the generation of ortho-PDX models. According to previous studies, colorectal tumors characterized by high ZEB2, EMT, stem cell traits and low proliferative index are more likely to belong to the CMS4 consensus molecular subtype of CRC (7, 42). Moreover, activation of the TGF-β pathway has been also reported to be associated with the CMS4 CRC subtype (42). The L6 CRC used in this study displayed several features of CMS4 tumors (ZEB2 overexpression, TGF-β activation, EMT, microsatellite stability) but lacks other features of CMS4 such as increased stromal content as compared to L5 and L7. On the other hand, the L6 tumor had KRAS mutation, which is typical of CMS3. Therefore, the molecular features presented by L6 (EMT/stemness/TGF-β/KRAS mutated) may indicate hybrid CMS3/CMS4 features that are particularly aggressive and prone to orthotopic engraftment. It may be speculated that tumors with the EMT/stemness/TGF-β/KRAS mutated signature may represent a class of “born to be bad” colorectal cancers with aggressive features and ability to metastatize at an early stage (43, 44). In line with this hypothesis, the L6 patient developed liver metastases within 18 months from surgery while L5 and L7 did not undergo metastatic progression (data not shown). The observations reported in this study have been performed on a very small number of cases and need to be supported by additional evidences. Despite the preliminary nature of our studies, we show that the ortho-PDX system is an effective and versatile tool to reproduce the features of metastatic CRC, encouraging a broader use of this model in translational CRC research.

## Data Availability Statement

The original contributions presented in the study are included in the article/**Supplementary Material**. Further inquiries can be directed to the corresponding author.

## Ethics Statement

The studies involving human participants were reviewed and approved by Sapienza-Policlinico Umberto I Ethical Committee (RIF.CE: 4107 17/10/2016; protocol n. 668/09, July 09, 2009; amended protocol 179/16, March 01, 2016). The patients/participants provided their written informed consent to participate in this study. The animal study was reviewed and approved by Animal Experimentation Committee of the Italian Ministry of Health (DM n. 292/2015 PR 23/4/2015).

## Author Contributions

MLDA and FF conceived the study and performed experiments. CN, DM and SV, provided essential technical services. AB provided genetic data collection and analysis. LC and FT provided clinical samples and expertise. EX performed experiments and provided clinical samples. PG and MB provided essential conceptual expertise. AZ conceived the study, supervised the project and wrote the manuscript. All authors contributed to the article and approved the submitted version.

## Funding

This work was supported by an Italian Association for Cancer Research (AIRC) Investigator Grant to AZ (AIRC IG 2017 Ref: 20744).

## Conflict of Interest

The authors declare that the research was conducted in the absence of any commercial or financial relationships that could be construed as a potential conflict of interest.

## Publisher’s Note

All claims expressed in this article are solely those of the authors and do not necessarily represent those of their affiliated organizations, or those of the publisher, the editors and the reviewers. Any product that may be evaluated in this article, or claim that may be made by its manufacturer, is not guaranteed or endorsed by the publisher.
