# Supplementary figures and images for: An Orthotopic Patient-Derived Xenograft (PDX) Model Allows the Analysis of Metastasis-Associated Features in Colorectal Cancer
Source: Front Oncol. 2022 Jun 28;12:869485. doi: 10.3389/fonc.2022.869485 (PMC9275818; doi:10.3389/fonc.2022.869485)

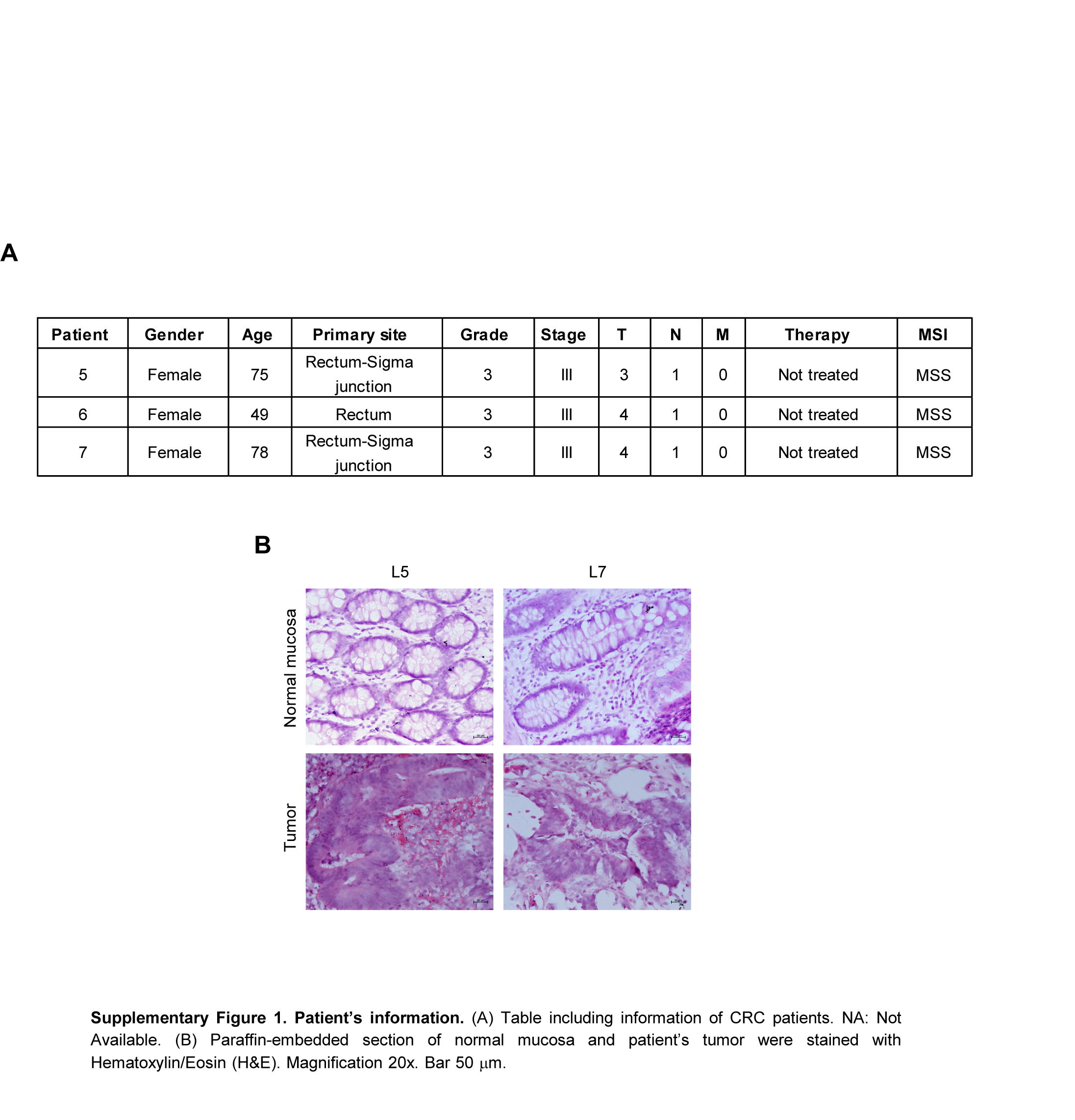

Supplement: Supplementary file 1 [file Image_1.tif]

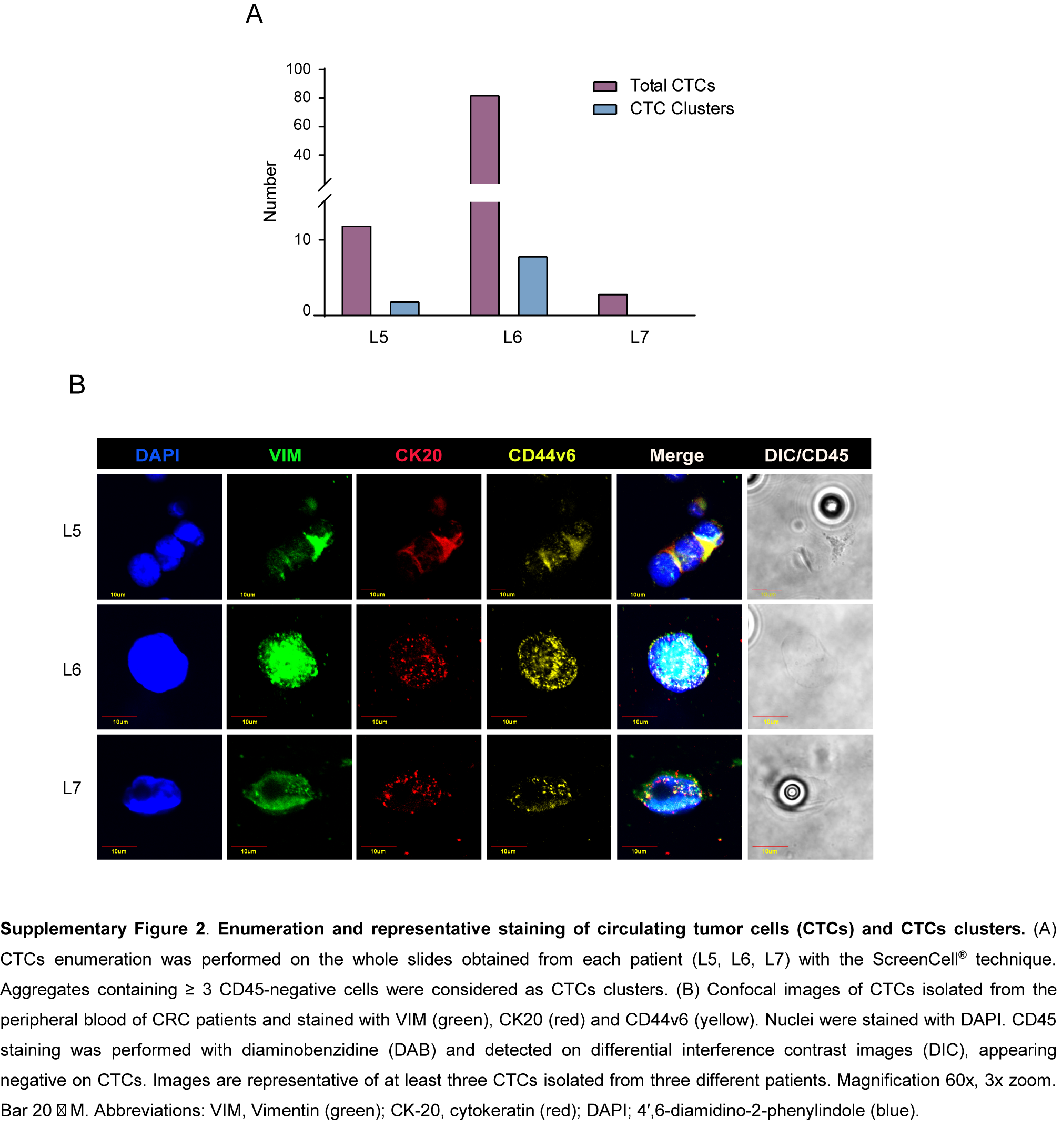

Supplement: Supplementary file 2 [file Image_2.tif]

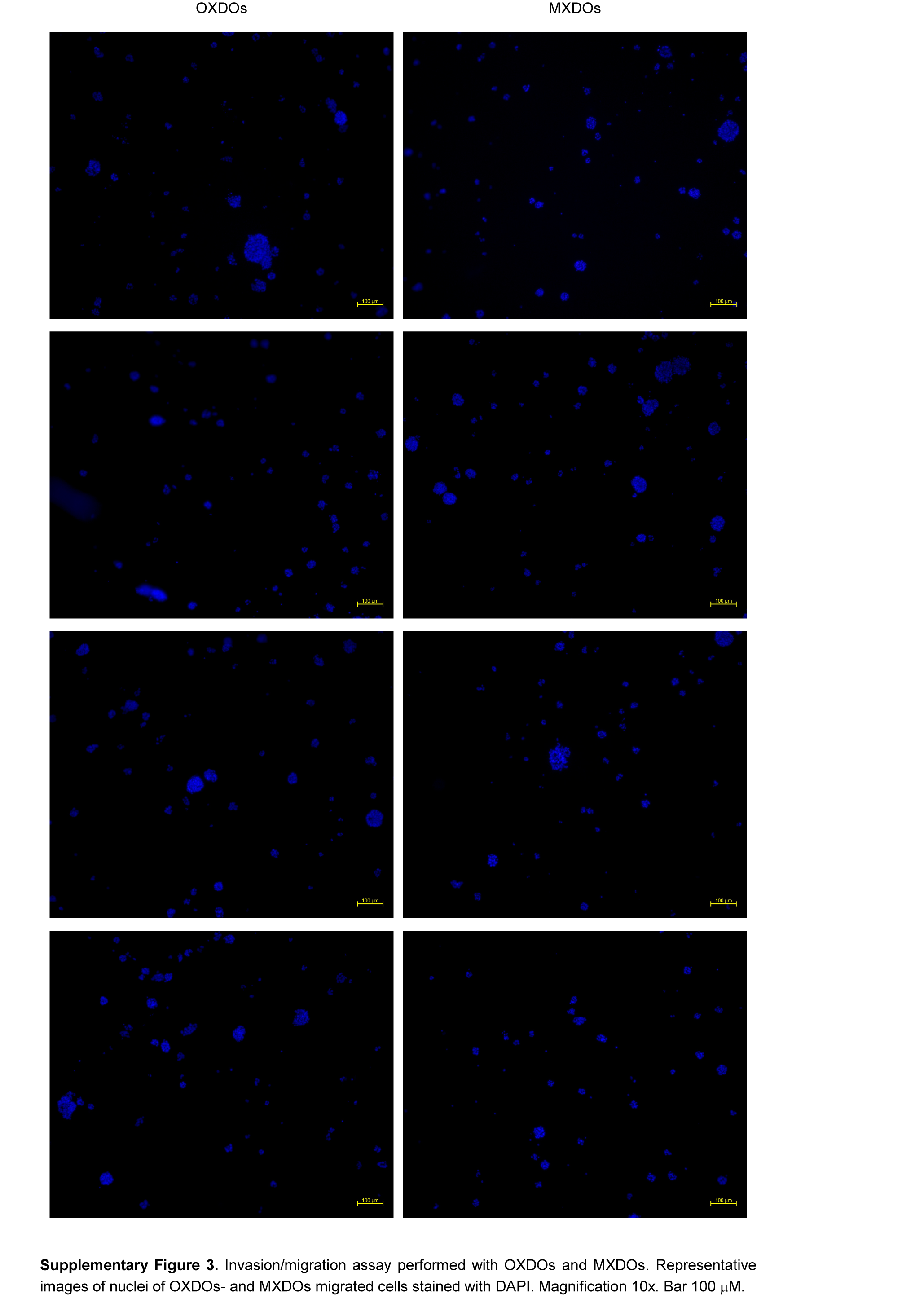

Supplement: Supplementary file 3 [file Image_3.tif]
